# Knowledge, attitudes, and practices toward COVID-19 among university students in Japan and associated factors: An online cross-sectional survey

**DOI:** 10.1371/journal.pone.0244350

**Published:** 2020-12-21

**Authors:** Asuka Hatabu, Xinhua Mao, Yi Zhou, Norihito Kawashita, Zheng Wen, Mikiko Ueda, Tatsuya Takagi, Yu-Shi Tian

**Affiliations:** 1 Graduate School of Pharmaceutical Sciences, Osaka University, Suita City, Osaka, Japan; 2 The Faculty of Psychology, Kobe Gakuin University, Kobe, Japan; 3 Graduate School of Science and Engineering Research, Kindai University, Higashiosaka City, Osaka, Japan; 4 Faculty of Science and Engineering, Waseda University, Shinjuku-ku, Tokyo, Japan; Leibniz Institute for Prevention Research and Epidemiology BIPS, GERMANY

## Abstract

The coronavirus disease (COVID-19) pandemic has greatly altered peoples’ daily lives, and it continues spreading as a crucial concern globally. Knowledge, attitudes, and practices (KAP) toward COVID-19 are related to individuals’ adherence to government measures. This study evaluated KAP toward COVID-19 among university students in Japan between May 22 and July 16, 2020, via an online questionnaire, and it further investigated the associated determining KAP factors. Among the eligible respondents (n = 362), 52.8% were female, 79.0% were undergraduate students, 32.9% were students whose major university subjects were biology-related, 35.4% were from the capital region, and 83.7% were Japanese. The overall KAP of university students in Japan was high. All respondents (100%) showed they possessed knowledge on avoiding enclosed spaces, crowded areas, and close situations. Most respondents showed a moderate or higher frequency of washing their hands or wearing masks (both at 96.4%). In addition, 68.5% of respondents showed a positive attitude toward early drug administration. In the logistic regressions, gender, major subjects, education level, nationality, residence, and psychological factors (private self-consciousness and extroversion) were associated with knowledge or attitudes toward COVD-19 (p < 0.05). In the logistic and multiple linear regressions, capital regions, high basic knowledge, high information acquisition, correct information explanations contributed positively to preventative action (p < 0.05). Non-capital regions, male gender, non-bio-backgrounds, high public self-consciousness, high advanced knowledge, incorrect information explanations, and high extroversion contributed negatively to self-restraint (p < 0.05). Moreover, self-restraint was decreasing over time. These findings clarify the Japanese university students’ KAP and the related factors in the early period of the COVID-19 pandemic, and they may help university managers, experts, and policymakers control the future spread of COVID-19 and other emerging infections.

## Introduction

The coronavirus disease (COVID-19) has become a global health concern. The World Health Organization characterized COVID-19 as a pandemic on March 11, 2020 [1]. As of November 13, 2020, the number of global confirmed cases and deaths has risen to over 52,657,000 and 1,291,000, respectively. In Japan, more than 113,600 infections and 1,800 deaths were confirmed [2].

Effective antivirals and vaccines are currently being developed, and effective therapeutic solutions have not been ultimately approved [3, 4]. Therefore, protecting citizens from new infections and health care institutions from using up capacities has become extremely important for all the countries. Many governments conducted lockdowns and interruption of citizens’ economic/social activities during rapid infection increases. These countermeasures were remarkable, while their effectiveness depended on the knowledge, attitudes, and preventative practices (KAP) toward COVID-19 among citizens, according to KAP theory and previous experiences [5, 6]. Meanwhile, countermeasures dramatically converted citizens’ lifestyles and daily behaviors, and thus change in the mental health, well-being, and psychological impacts related to COVID-19 have also been highlighted and investigated [7, 8]. For example, a large-scale international survey to analyze citizens’ mental well-being at the onset of the COVID-19 pandemic [9] and a large-scale international survey to evaluate the students’ well-being have been conducted [10, 11].

In Japan, the government issued several foundational policies for preventing and controlling COVID-19, including an emergency declaration for Tokyo, Kanagawa, Saitama, Chiba, Osaka, Hyogo, and Fukuoka on April 8, 2020, and later for the whole country until May 25, 2020 [12]. Although the emergency declaration was significant to control the rapid increase of COVID-19 infections, it can not last long due to socioeconomic losses. After lifting of declaration, increases in COVID-19 infections started, including those in the young generation [13].

At the onset of COVID-19, a survey on Japanese citizens’ behavioral changes and preparedness against COVID-19 conducted by Muto *et al*. revealed that being younger was among the factors associated with reluctance to follow prevention measures [14]. Owing to the explanation and awareness that older individuals are at a highest risk of becoming severely ill or passing away [15, 16], there is a possibility that the young Japanese generation has not paid enough attention to COVID-19. Although later research stated that “COVID-19 does not spare young people” [17, 18], there is still a risk that the young generation will not undertake precaution measures as necessary. Moreover, young asymptomatic cases have the possibility of spreading viruses to the high-risk population. Therefore, the young Japanese generation’s KAP that influence the compliance with countermeasures should be evaluated.

Japan’s 2019 university/junior college entrance rate was 58.1% [19], suggesting that university students account for most young individuals. University students have higher economic independence, higher autonomy, and are less dependent on parents than high school students or those with a lower level of education [20]. High school students or those with a lower level of education are more likely to follow their parents’ lead and further obey the government countermeasures. In contrast, intelligent university students can judge their surroundings and exhibit behaviors based on their judgment, especially those who live separately from their families. Meanwhile, university students engage in vigorous activities, such as academic activities, sports clubs, and part-time jobs, and for this reason, they have more opportunities to get in contact with others. These aspects increase the importance of analyzing the university students’ KAP in Japan, as reported from other countries [21, 22]. Knowing the states of KAP toward COVID-19 among university students and further analyzing the KAP factors can play vital roles in planning and confirming the countermeasures for the young generation in the COVID-19 prevention.

So far, to the best of our knowledge, there is a survey that investigated Japanese university students’ awareness and actions toward COVID-19 [23], but the knowledge of COVID-19 and factors influencing KAP have not been evaluated yet. Previous surveys on KAP and well-being in other countries showed that citizens and university students had related high levels of knowledge about COVID-19 and displayed positive attitudes and low-risk practices. Differences in gender, age, education level, and major fields of studies/backgrounds affect the levels of knowledge, the practices such as appropriated hygiene and social distancing behaviors, and sometimes psychological health (i.e., anxiety, depression, etc.) [5, 21, 24–27]. Whether these factors affect Japanese university students’ KAP toward COVID-19 has not been studied. Moreover, because most studies only focus on practice, not much is known what affects the extent of knowledge and attitude. This information should be paramount in improving university students’ knowledge and attitudes.

Besides the factors listed above, psychological factors are also assumed to be crucial to university students’ behaviors and practices. University students are independent of their families, forming their own identities [28]. They are very concerned about how they present themselves and how people see them. In an emergency such is the COVID-19 pandemic, in which behaviors are strictly restrained, one’s behavior is more frequent based on the viewpoints of their own and others. Self-consciousness [29] is considered to significantly influence young people’s behavior as a factor determining their behavior. Another critical factor is personality. It can be assumed that the extroverted nature of actively interacting with others determines university students’ range of action.

This study conducted an online survey to evaluate KAP toward COVID-19 among university students in Japan. Meanwhile, we attempted to examine KAP’s differences related to the factors such as gender, education level, nationality, residence, responders’ major, and their psychological characteristics (i.e., extroversion and self-consciousness) and the relationship between KAP and these factors.

## Materials and methods

### Study design, participants, and data collection

This cross-sectional study administered an anonymous survey using a questionnaire constructed using Google Forms. Participants’ inclusion criteria were university/college/junior college students who lived in Japan and could read and understand Japanese or English. Due to the emergency and particular period, we adopted a convenience sampling. We distributed our online survey form together with a quick response (QR) code via direct deliberation in laboratories, online lectures and lecture homepages, university club mailing lists, and social networks (Facebook, Twitter, Line, and WeChat). The answer procedures, the voluntary nature of participation, and anonymity declaration within the explanation of informed consent were presented on the questionnaire’s top page. Responders answered the questionnaire via their internet surroundings. Data were collected from May 22 to July 16, 2020, crossing the emergency declaration lift at 8 weeks, which is the earliest time after receiving an ethical review approval.

### Questionnaire design

We designed a questionnaire (**S1 File in S1 Data**) so as to have seven categories: demographic information, knowledge about COVID-19 and virology (Knowledge), approach and frequency of obtaining information and comprehension level (Information), social behaviors and actions (Behavior), personal-psychological aspects (Psychological aspects), change in awareness before and after the “declaration of emergency,” (Awareness) and concerns about online lectures (Lecture). The questionnaire was designed in Japanese with an English translation.

Demographic information included gender, respondents’ majors (i.e., “humanities subjects” OR “medicine, dentistry, or pharmaceutical science” OR “science subjects (related to biological subjects)” OR “science subjects (not related to biological subjects”), grade, age, nationality, residence, and so forth. Awareness included two questions asking whether there was a change in awareness before and after the emergency declaration and the percentage of change. Psychological aspects were designed based on two instruments: extroversion from a short form of the Japanese Big-Five Scale (5-point Likert scale) [30] and public/private self-consciousness scales from the self-consciousness scale for Japanese (7-point Likert scale) [31]. Knowledge, Information, Behavior, and Lecture were self-designed as 6-point Likert scales (1 meaning “disagree at all” to 6 meaning “strongly agree”) according to several questionnaires for infectious diseases [32–34].

### Data preprocessing

Typos were corrected. Different terminology for the same nationality or residences were unified. Inconsistent responses were corrected in an interpretable direction. For example, when the “self-defense change” was answered as “increased,” but its percentage was answered with 0, we corrected the answer to “no change.” To align the positive with the desired direction and provide a concise view, we exchange responses to reverse questions (**S1 File in S1 Data**) using the largest scale + 1—original value.

Scales for each category were validated using factor analysis, and variables for the following analyses were generated. The number of factors was decided by Wayne Velicer’s minimum average partial criterion (MAP) or Bayesian information criterion (BIC). If the number of factors was not 1, the Promax rotation was conducted. Questions with the magnitude of loadings < 0.4 were removed. Variables were created from the subscale scores obtained from the mean of the questions within factors. Finally, 10 continuous variables indicating *psychological aspects* and KAP *(extroversion*, *public self-consciousness*, *private self-consciousness*, *basic knowledge*, *advanced knowledge*, *info acquisition*, *info explanation*, *info anxiety*, *self-restraint*, *and preventative action)* with acceptable internal consistencies (Cronbach’s α = 0.73 ~ 0.92) were obtained (**S2 File in S1 Data**).

For the following subgroup comparisons, based on the response time recorded, we created a binary variable *response time* (“early” responses obtained in 0–4 weeks vs. “late” responses obtained in 5–8 weeks). Several variables were organized into binary variables: *major subject* (“bio-backgrounds”: “medicine, dentistry, or pharmaceutical science” or “science subjects (related to biological subjects)” is selected vs. “non-bio-backgrounds”: others, *education level* (“undergraduate” vs. “graduate or above”), *nationality* (“Japanese” vs. “others”), and *residence* (“capital region” vs. “others”). Psychological aspects (*extroversion*, *public self-consciousness*, *private self-consciousness*) were also converted into binary values as “high” or “low.”

### Data analysis

#### Descriptive statistics

To directly evaluate KAP toward COVID-19 among university students in Japan, the responses to the questions were aggregated, and the extent and magnitude of KAP were confirmed.

#### Subgroup comparisons

To examine responses to what questions are different caused by the factors (e.g., response time, gender, major subject, education level, nationality, residence and psychological aspects [extroversion, public self-consciousness, and private self-consciousness]) we carried out subgroup comparisons.

Subgroup comparisons for all the questions were conducted to determine if there were any differences in responding to questions within subgroups. The normality and homoscedasticity of data were tested by the Shapiro–Wilk and *F*- tests. If data were non-normally distributed and homoscedastic, differences were tested using the Mann–Whitney method. Brunner–Munzel method was used to test the significance between two groups of non-normal but heteroscedastic data. The Bonferroni method was adopted for multiple comparisons. The subgroups were those generated in data preprocessing.

#### Logistic regression

Logistic regression models were constructed to evaluate whether the previously reported important variables from other countries’ surveys and psychology aspects mentioned above influence KAP toward COVID-19 among Japanese university students.

KAP outcomes were generated from factor analysis, and in logistic regression models were used as binary outcomes. Here, we created binarization (high/low or safe/unsafe) of basic knowledge, advanced knowledge, info acquisition, info explanation, info anxiety, self-restraint, and preventative action as outcomes. For the former four outcomes, explanatory variables used are response time, gender, major subject, education level, nationality, residence, and psychological aspects (extroversion, public self-consciousness, and private self-consciousness). For the last two outcomes indicating the actions, continuous variables of basic knowledge, advanced knowledge, info acquisition, info explanation, info anxiety, and self-restraint were further added, as we also want to confirm whether the knowledge and attitude to information influence the practices.

#### Multiple linear regression

Multiple linear regression (MLR) models were constructed to further confirm the factors of practices further. The outcomes were set to be binary in the above logistic regression, while in MLR, the outcomes were used as continuous values. Therefore, the MLR models were quantitative.

The MLR models for *self-restraint* and *preventative action* were constructed. The explanatory variables used were the same as above. Determinant factors were decided when Akaike’s Information Criterion (AIC) reached a minimum. Data were first normalized, and the variance inflation factor (VIF) was also calculated to confirm multicollinearity. The regression models’ predictive power was assessed by the mean *R*^2^, which is calculated from five-fold cross validation by repeating 50 times (a machine-learning approach). A significant level of 0.1 was adopted.

#### Software and package versions

All the analyses were conducted using R (version 3.6.2), RStudio (1.2.5033), Mephas Web (2020–01) [35], and R packages psych (1.9.11), coin (1.3–1), stats (3.6.2), lawstat (3.4), glm (3.6.2), lm (3.6.2), bestglm (0.33), car (3.0–8), and caret (6.0–86).

### Ethics

The Ethics Committee of Waseda University and the Graduate School of Pharmaceutical Sciences of Osaka University approved this study (2020-HN005 and Yakuhito2020-5). Informed consent was obtained from each participant on the first page of the questionnaire.

## Results

### Demographic characteristics

A total of 362 participants (female 52.8%) were included in the analyses after one participant’s response was removed due to the invalidate residence input (Table 1). The age of the participants was 20.8 ± 3.5 years. Students whose majors were biology related (bio-backgrounds) accounted for 32.9%, undergraduate students for 79.0%, Japanese students 83.7%, and participants from capital region residence 35.4%.

**Table 1:** The baseline information of participants.

	Categories	N = 362	
		n/mean	%/SD
*Gender*	female	191	52.8
	male	171	47.2
*Age*	-	20.8	3.5
*Major subjects*	non-bio-backgrounds	243	67.1
	bio-backgrounds	119	32.9
*Education level*	undergraduate	286	79.0
	graduate or above	76	21.0
*Nationality*	Japanese	303	83.7
	others	59	16.3
*Residence*	capital region	128	35.4
	others	234	64.6
*Online lecture experience*	no	25	6.9
	yes	337	93.1
*Change of awareness*	increased	260	71.8
	lowered	2	0.6
	no change	100	27.6

### Overall results showed that respondents were inclined toward safety and good health

**Fig 1:**
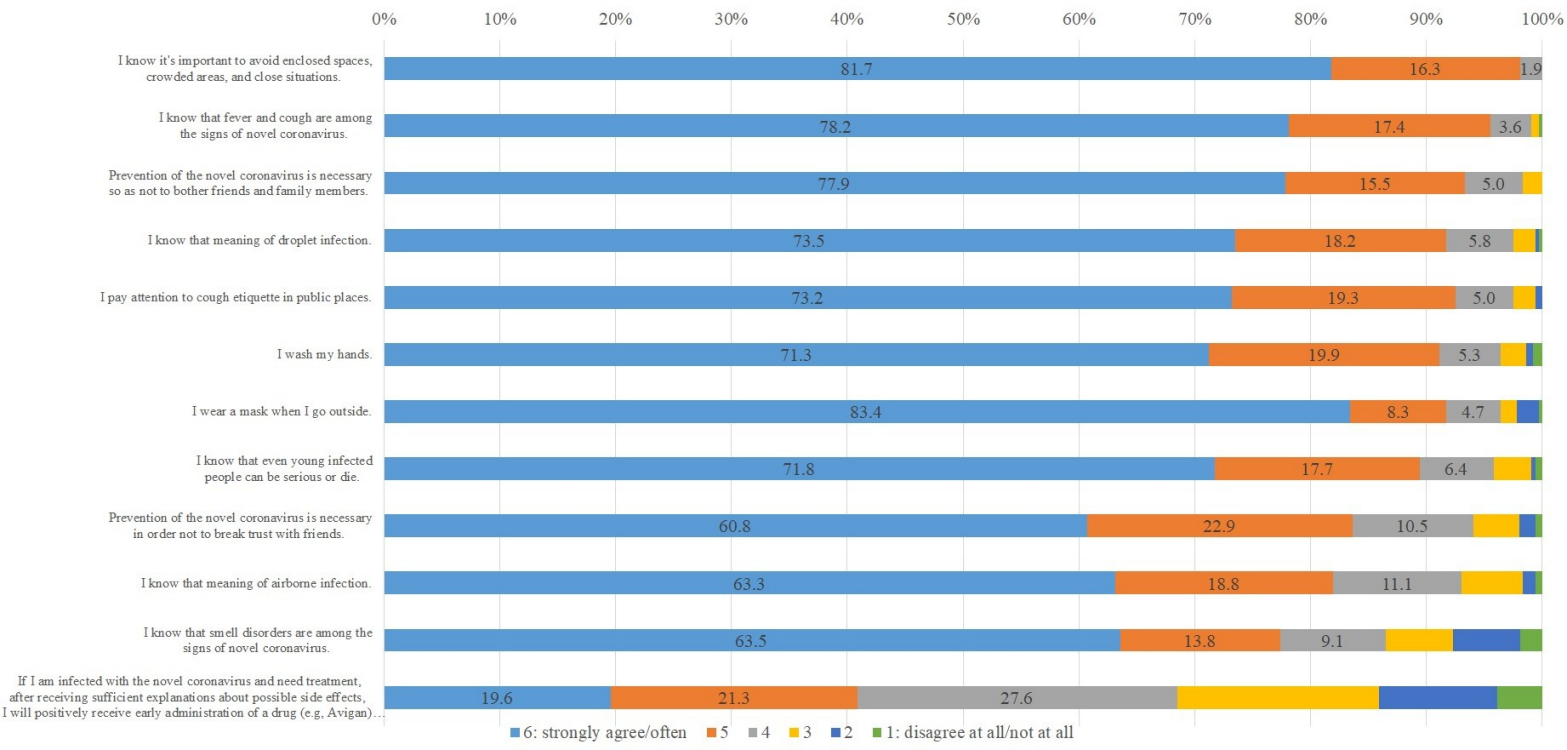
Questions obtaining high safety and good health answers (n = 362).

The overall results showed that the respondents were inclined toward safety and good health. Proportions of responses larger than the theoretical median of 3.5 varied from 24.6% to 100% (**S3 File in S1 Data**). The highest response score was to “I know it’s important to avoid enclosed spaces, crowded areas, and close situations.” No response was less than the theoretical median, suggesting a deep understanding of the Japanese government’s advisement of 3Cs [36] (**Fig 1**). High scores were also obtained in the knowledge or awareness of the infectious routes, vital signs, the severity of the virus, preventative measures (e.g., handwashing, mask-wearing). Additionally, 68.5% of respondents showed a positive attitude toward early drug administration (e.g., Avigan) (**Fig 1**).

### Differences detected among subgroups

The significant differences among subgroups were extracted (**S4 File in S1 Data**). Late response time (5–8 weeks) showed significant lower medians in the questions related to the tension toward COVID-19, suggesting that with time, although the basic knowledge has increased, tension has been eased. For gender differences, females showed conservative/safer attitudes than males in the stance to close bars and go to university. Compared to non-bio-backgrounds, bio-backgrounds showed a higher level of advanced knowledge as expected. Surprisingly, non-bio-backgrounds showed a higher score on the opinion, “I think that I can naturally heal without medical care such as hospitalization even the novel coronavirus infects me (reversed),” suggesting a safer consideration on the COVID-19 infection.

Similarly, for education level, students at the graduate level or above had significantly higher advanced knowledge, stronger willingness to accept anxious news, and slightly higher satisfaction about online lectures and assignments. In terms of nationality, Japanese students knew more basic knowledge and were more sensitive to the emergency declaration. In contrast, international students knew more advanced knowledge and had a stronger willingness to accept anxious news and correct information explanations. When considering the residence, students living in the capital region showed stronger self-restraint and acted more safely than others. From the psychological aspects, more active information collection was detected in the high extroversion and the high private self-consciousness groups.

### Factors influencing university students’ knowledge levels

Logistic regressions were conducted to explore the determinant factors on high basic knowledge and advanced knowledge (**Table 2**).

**Table 2:** Logistic regression models for university students’ knowledge levels.

**a) Logistic regression model for basic knowledge**							
	**Coefficients**	**S.E.**	**Wald Z**	**Odds Ratio**	**95% Lower CL**	**95% Upper CL**	**p-value**
Constant	-1.029	0.794	-1.297	0.357	0.074	1.679	0.195
*Response time* late	-0.229	0.282	-0.811	0.796	0.454	1.376	0.417
*Gender* male	-0.453	0.237	-1.912	0.636	0.398	1.009	0.056 *
*Major subject* non-bio-backgrounds	-0.619	0.291	-2.132	0.538	0.303	0.947	0.033 **
*Education level* undergraduate	-0.812	0.393	-2.064	0.444	0.200	0.942	0.039 **
*Nationality* Japanese	1.202	0.458	2.626	3.327	1.381	8.379	0.009 ***
*Residence* others	-1.032	0.289	-3.566	0.356	0.200	0.624	0.000 ***
*Extroversion*	0.242	0.127	1.901	1.273	0.994	1.639	0.057 *
*Public Self-Consciousness*	0.025	0.079	0.315	1.025	0.877	1.197	0.753
*Private Self-Consciousness*	0.305	0.096	3.185	1.356	1.127	1.642	0.001 ***
**b) Logistic regression model for advanced knowledge**							
	**Coefficients**	**S.E.**	**Wald Z**	**Odds Ratio**	**95% Lower CL**	**95% Upper CL**	**p-value**
Constant	2.464	0.904	2.727	11.746	2.055	71.786	0.006 ***
*Response time* late	-0.038	0.314	-0.120	0.963	0.521	1.787	0.904
*Gender* male	0.590	0.267	2.208	1.804	1.074	3.069	0.027 **
*Major subject* non-bio-backgrounds	-3.010	0.382	-7.874	0.049	0.023	0.101	0.000 ***
*Education level* undergraduate	-0.837	0.454	-1.843	0.433	0.173	1.036	0.065 *
*Nationality* Japanese	-1.041	0.509	-2.045	0.353	0.128	0.955	0.041 **
*Residence* others	-1.202	0.334	-3.595	0.301	0.153	0.570	0.000 ***
*Extroversion*	0.250	0.143	1.754	1.284	0.973	1.704	0.079 *
*Public Self-Consciousness*	-0.275	0.092	-2.99	0.759	0.632	0.907	0.003 ***
*Private Self-Consciousness*	0.440	0.113	3.901	1.553	1.250	1.948	0.000 ***

* p < 0.1

** p < 0.05

*** p < 0.01

For basic knowledge, gender, major subject, education level, nationality, residence, extroversion, and private self-consciousness were significant determinant factors (**Table 2A**). The odds of Japanese students gaining basic knowledge was 3.33 times greater than others (international students), which strongly implies that Japanese students gained more basic knowledge than international students. The odds ratios (ORs) of extroversion and private self-consciousness were also >1, suggesting that extroverts and individuals with high private self-consciousness were likely to possess more basic knowledge than others. The ORs of gender, major subject, education, and residence were <1, suggesting that females, with bio-backgrounds, at the level of graduate or above and in the capital region could positively influence the acquisition of basic knowledge.

For advanced knowledge, except for response time, all the explanatory variables were significant (**Table 2B**). Bio-backgrounds, living in the capital region, low public self-consciousness, and high private self-consciousness strongly positively influence the acquisition of advanced knowledge.

### Factors influencing university students’ attitudes

The university students’ attitudes to COVID-19 were assessed considering the frequency and activities of information acquisition (info acquisition), the correct explanation of the information (info explanation), and willingness to collect anxiety information (info anxiety) (**Table 3**).

**Table 3:** Logistic regression models for university students’ attitudes.

**a) Logistic regression model for frequent or active information acquisition (Info acquisition)**							
	**Coefficients**	**SE**	**Wald Z**	**Odds Ratio**	**95% Lower CL**	**95% Upper CL**	**p-value**
Constant	-2.327	0.798	-2.914	0.098	0.020	0.457	0.004 ***
*Response time* late	-0.081	0.278	-0.291	0.922	0.532	1.584	0.771
*Gender* male	0.197	0.233	0.847	1.218	0.773	1.926	0.397
*Major subject* non-bio-backgrounds	0.170	0.286	0.593	1.185	0.676	2.078	0.553
*Education level* undergraduate	-0.316	0.371	-0.851	0.729	0.348	1.498	0.395
*Nationality* Japanese	0.444	0.436	1.018	1.559	0.662	3.689	0.309
*Residence* others	-0.143	0.281	-0.511	0.866	0.498	1.502	0.609
*Extroversion*	0.255	0.125	2.042	1.290	1.012	1.652	0.041 **
*Public Self-Consciousness*	0.140	0.078	1.788	1.150	0.987	1.343	0.074 *
*Private Self-Consciousness*	0.216	0.092	2.338	1.241	1.037	1.490	0.019 **
**b) Logistic regression model for correct information explanation (Info explanation)**							
	**Coefficients**	**SE**	**Wald Z**	**Odds Ratio**	**95% Lower CL**	**95% Upper CL**	**p-value**
Constant	0.170	0.790	0.215	1.185	0.252	5.631	0.830
*Response time* late	-0.305	0.277	-1.104	0.737	0.426	1.264	0.270
*Gender* male	-0.607	0.234	-2.597	0.545	0.343	0.859	0.009 ***
*Major subject* non-bio-backgrounds	0.328	0.282	1.163	1.388	0.798	2.417	0.245
*Education level* undergraduate	-0.033	0.368	-0.091	0.967	0.471	2.004	0.928
*Nationality* Japanese	-0.074	0.442	-0.168	0.928	0.385	2.193	0.866
*Residence* others	-0.557	0.284	-1.960	0.573	0.326	0.996	0.050 *
*Extroversion*	0.204	0.124	1.642	1.226	0.962	1.568	0.101
*Public Self-Consciousness*	-0.157	0.080	-1.952	0.855	0.728	0.999	0.051 *
*Private Self-Consciousness*	0.190	0.093	2.040	1.210	1.009	1.457	0.041 **
**c) Logistic regression model for disagreement with anxious news and countermeasures (Info anxiety)**							
	**Coefficients**	**SE**	**Wald Z**	**Odds Ratio**	**95% Lower CL**	**95% Upper CL**	**p-value**
Constant	-1.796	0.796	-2.256	0.166	0.034	0.778	0.024 **
*Response time* late	-0.585	0.287	-2.036	0.557	0.313	0.968	0.042 **
*Gender* male	0.242	0.232	1.041	1.273	0.808	2.012	0.298
*Major subject* non-bio-backgrounds	-0.378	0.290	-1.302	0.686	0.387	1.207	0.193
*Education level* undergraduate	-0.005	0.378	-0.014	0.995	0.464	2.065	0.989
*Nationality* Japanese	1.461	0.452	3.233	4.308	1.813	10.751	0.001 ***
*Residence* others	0.166	0.278	0.596	1.180	0.685	2.040	0.551
*Extroversion*	0.218	0.125	1.744	1.243	0.975	1.592	0.081 *
*Public Self-Consciousness*	0.030	0.079	0.375	1.030	0.881	1.204	0.708
*Private Self-Consciousness*	0.088	0.093	0.952	1.092	0.911	1.311	0.341

* p < 0.1

** p < 0.05

*** p < 0.01

For the frequency and activity of information acquisition, only psychology aspects showed significance and were positively associated with the outcome (**Table 3A**). The determinant factors for the correct explanation of the information were female, residence, public self-consciousness, and private self-consciousness (**Table 3B**). Incorrect explanations may affect students’ protection and further increase their infection risk. For the willingness to collect anxiety information, response time, nationality, and extroversion are significant. International students and low extroversion are more likely to receive information regardless of whether it can make people anxious (**Table 3C**). Notably, the OR (late vs. early responses) was 0.56, suggesting that more students were willing to receive anxious news and countermeasures with time passing.

### Factors influencing university students’ preventative practices and behaviors

Preventative practices and behaviors included self-restraint and preventative action. Two logistic regression models for these behavior terms were constructed using the variables mentioned above and variables of knowledge and information as explanatory variables (**Table 4**).

**Table 4:** Logistic regression models for university students’ preventative practices and behaviors.

**a) Logistic regression model for strong self-restraint (Self-restraint)**							
	**Coefficients**	**SE**	**Wald Z**	**Odds Ratio**	**95% Lower CL**	**95% Upper CL**	**p-value**
Constant	3.637	0.994	3.660	37.972	5.664	281.094	0.000 ***
*Response time* late	-1.383	0.326	-4.238	0.251	0.130	0.468	0.000 ***
*Gender* male	-0.577	0.264	-2.190	0.561	0.333	0.938	0.028 **
*Major subject* non-bio-backgrounds	-0.483	0.369	-1.310	0.617	0.297	1.265	0.190
*Education level* undergraduate	0.126	0.426	0.296	1.135	0.493	2.641	0.767
*Nationality* Japanese	0.421	0.537	0.785	1.524	0.532	4.398	0.432
*Residence* others	-1.125	0.329	-3.418	0.325	0.168	0.614	0.001 ***
*Extroversion*	-0.279	0.140	-1.993	0.757	0.573	0.993	0.046 **
*Public Self-Consciousness*	-0.132	0.093	-1.415	0.877	0.729	1.051	0.157
*Private Self-Consciousness*	-0.155	0.107	-1.445	0.856	0.693	1.056	0.149
*Basic knowledge*	0.170	0.283	0.599	1.185	0.679	2.065	0.549
*Advanced knowledge*	-0.233	0.321	-0.728	0.792	0.421	1.485	0.467
*Info acquisition*	0.164	0.281	0.585	1.179	0.68	2.052	0.559
*Info explanation*	1.637	0.264	6.189	5.139	3.089	8.730	0.000 ***
*Info anxiety*	-0.488	0.266	-1.837	0.614	0.363	1.031	0.066 *
**b) Logistic regression model for safe preventative action (Preventative action)**							
	**Coefficients**	**SE**	**Wald Z**	**Odds Ratio**	**95% Lower CL**	**95% Upper CL**	**p-value**
Constant	-3.736	0.951	-3.926	0.024	0.004	0.148	0.000 ***
*Response time* late	0.435	0.305	1.425	1.545	0.851	2.824	0.154
*Gender* male	-0.160	0.260	-0.615	0.852	0.511	1.418	0.538
*Major subject* non-bio-backgrounds	0.121	0.362	0.334	1.128	0.555	2.302	0.739
*Education level* undergraduate	0.595	0.409	1.455	1.813	0.819	4.099	0.146
*Nationality* Japanese	0.061	0.496	0.123	1.063	0.399	2.821	0.902
*Residence* others	-0.786	0.315	-2.492	0.456	0.244	0.841	0.013 **
*Extroversion*	0.103	0.139	0.739	1.108	0.844	1.458	0.460
*Public Self-Consciousness*	0.115	0.088	1.305	1.121	0.944	1.334	0.192
*Private Self-Consciousness*	0.170	0.103	1.654	1.185	0.971	1.453	0.098 *
*Basic knowledge*	0.647	0.270	2.395	1.910	1.124	3.247	0.017 **
*Advanced knowledge*	0.434	0.318	1.365	1.543	0.830	2.897	0.172
*Info acquisition*	0.678	0.266	2.550	1.970	1.170	3.327	0.011 **
*Info explanation*	1.127	0.256	4.401	3.087	1.878	5.134	0.000 ***
*Info anxiety*	0.160	0.263	0.610	1.174	0.700	1.967	0.542

* p < 0.1

** p < 0.05

*** p < 0.01

For the self-restraint, response time, gender, residence, extroversion, info explanation, and info anxiety, there was statistical significance (**Table 4A**). Female, living in capital region, and low extroversion show a relatively safer self-restraint. The self-restraint is attenuated compared to early responses. Moreover, we found that information explanation (info explanation) positively, and the anxiety of information (info anxiety) negatively affected the self-restraint. For the preventative action, residence, private self-consciousness, basic knowledge, info acquisition, and info anxiety were significant (**Table 4B**).

We also applied MLR when treating self-restraint and self-action as continuous values. After variable selection using AIC, important variables remained (**Table 5**). The predictive abilities (*R*^2^) for the models were 0.45 and 0.34, respectively. Nested cross-validation revealed similar predictive abilities.

**Table 5:** AIC selected MLR models for university students’ preventative practices and behaviors.

**a) AIC selected MLR model for self-restraint (Self-restraint)**					
	**Estimate**	**SE**	**t-value**	**p-value**	**VIF**
Intercept	0.817	0.190	4.297	0.000 ***	-
*Response time* late	-0.552	0.099	-5.566	0.000 ***	1.431
*Gender* male	-0.207	0.084	-2.454	0.015 *	1.131
*Major subject* non-bio-background	-0.411	0.119	-3.448	0.001 ***	1.991
*Nationality* Japanese	0.241	0.137	1.756	0.08 .	1.635
*Residence* others	-0.446	0.101	-4.430	0.000 ***	1.477
*Extroversion*	-0.074	0.041	-1.781	0.076 .	1.081
*Public Self-Consciousness*	-0.116	0.042	-2.771	0.006 **	1.106
*Advanced knowledge*	-0.122	0.050	-2.470	0.014 *	1.560
*Info explanation*	0.545	0.041	13.156	0.000 ***	1.089
*Info anxiety*	-0.072	0.042	-1.695	0.091 .	1.136
**b) AIC selected MLR model for preventative action (Preventative action)**					
	**Estimate**	**SE**	**t-value**	**p-value**	**VIF**
Intercept	0.138	0.073	1.887	0.060 .	-
*Residence* others	-0.213	0.091	-2.333	0.020 *	1.035
*Private Self-Consciousness*	0.074	0.045	1.656	0.099 .	1.082
*Basic knowledge*	0.293	0.047	6.207	0.000 ***	1.205
*Info acquisition*	0.219	0.048	4.571	0.000 ***	1.240
*Info explanation*	0.268	0.045	5.978	0.000 ***	1.086
*Info anxiety*	0.065	0.045	1.465	0.144	1.072

a) Signif. codes: 0 ‘***’ 0.001 ‘**’ 0.01 ‘*’ 0.05 ‘.’ 0.1 ‘ ‘ 1

R-squared: 0.447

Predictive ability

**R-squared (training set):** 0.450 ± 0.032; R-squared (cross validation (mean)): 0.373 ± 0.042; R-squared (test set): 0.374 ± 0.134

b) Signif. codes: 0 ‘***’ 0.001 ‘**’ 0.01 ‘*’ 0.05 ‘.’ 0.1 ‘ ‘ 1

R-squared: 0.344

**Predictive ability:**

R-squared (training set): 0.345 ± 0.020; R-squared (cross validation (mean)): 0.278 ± 0.037; R-squared (test set): 0.297 ± 0.103

The *self-restraint* model revealed that correct information explanation and Japanese nationality were associated with strong self-restraint **(Table 5A)**. On the other hand, male, non-bio-backgrounds, living in the non-capital region, high advanced knowledge, and unwillingness to receive anxious information, and extroversion negatively influenced the self-restraint. The model for *preventative action* was constructed using less explanatory variables. The coefficients of private self-consciousness, basic knowledge, and variables about information were positive, and that of residence in the non-capital region were negative **(Table 5B)**. The results generally consist of logistic regressions: Students living in the capital region, having more basic knowledge, frequently inquiring information, correctly explaining information, and with high private self-consciousness tend to act more safely.

## Discussion

### University students in Japan exhibited a relatively high level of basic knowledge and awareness

University students in Japan belong to a representative group of the young generation. The outbreak of unprecedented COVID-19 pandemic directly impedes their daily lives and activities. Compared to the employees and other adult populations, university students exhibit less financial independence but have more free time and broader activities. Meanwhile, university days are the most crucial period in forming one’s self-will, and university students are much more likely to act on their self-judgment than other students. To control the spread of infections, governments provided guidelines and countermeasures, and KAP influences adherence to them. Since university students belong to a separate population according to the aspects above, we first evaluated their KAP toward COVID-19.

Overall, we found a high level of basic knowledge on COVID-19 and control measures among university students in Japan. For example, with regard to the question about avoiding enclosed spaces, crowded areas, and close situations (three Cs) [36], all the responses were no less than the theoretical median, and the frequency of handwashing and mask-wearing no less than the theoretical median were both 96.4%, indicating university students clearly understand the importance of avoiding the “overlapping three Cs” and the basic protective methods. These results were in line with the previous survey result in February according to which approximately 83.8% of Japanese citizens always or sometimes conducted hand hygiene [37]. These results could be a possible reason for effective control of the infection in the early period after the emergency was declared. Several similar surveys from other countries also showed high levels of university students’ KAP toward COVID-19. Our results agree with these previous surveys and compensate for the lack of Japanese data. For example, considering the view on mask-wearing, approximately 52.1% of Jordan’s university students [21] and 98.0% of Chinese (Wuhan region) university students [5] wore a facemask when leaving home, according to their responses from March and January, respectively, and 86.9% of Indonesian undergraduate students wore masks frequently when in a crowded place [22], according to their responses collected in April and May. Our results showed that the rate of frequent mask-wearing in Japanese students was 96.4%, which is still relatively high compared with the global results. Although it has not been proven, a commentary published in April hypothesized that Japanese culture, which is inherently suited for social distancing and face mask use, prevents viral spread [38]. This may be the reason why our results were relatively high.

Students with high education levels and bio-backgrounds were found to have more advanced knowledge about viruses, vaccines, and drug targets. Interestingly, international students were prone to having more advanced knowledge, while Japanese students more basic knowledge. For some basic knowledge, such as diarrhea and taste disorders being symptoms of COVID-19, Japanese and international students showed slight differences, indicating that the route of obtaining basic knowledge may be different. Therefore, particular education/information about Japan’s countermeasures for international students may be necessary. Other KAP studies in global did not separate knowledge into basic and advanced. Therefore, our results provide novel information. Moreover, we found that females had more basic knowledge and explained the information more correctly than males, which agrees with previous studies in other countries showing that females have higher knowledge about COVID-19 and a proper attitude [39, 40].

### Students living in the capital region conduct themselves with strong self-restraint and safe preventative action, however, there is a decrease in self-restraint over time

The behaviors of students were evaluated using self-restraint and preventative action. For all the models, the residence was a significant factor, and students living in the capital region exhibited safer behaviors when compared to other regions. Until now, infections have been most extensive in Tokyo, enhancing the awareness of capital region residents. Therefore, behaviors took a safer direction. A survey targeting Indonesian undergraduate students showed that rural students showed significantly higher KAP than those living in cities, which seems to be in disagreement with the current results. However, we compared the capital region and others in Japan, and the severity of the infection situation was different, making students living in the capital region higher KAP toward COVID-19.

One noteworthy observation was that the logistical regressions and MLR showed the response time to be significant for self-restraint, indicating a decrease in self-restraint over time.

### Psychological aspects influence students’ behaviors

Behavior patterns of individuals differ depending on their personality. “Extroversion” and “introversion” have been terms widely used, and they depend on how people direct their energy, that is, externally or internally [41]. Extroverts tend to be more interested in the outside world and the decision-making process of things. They follow the “environment” when it comes to directions and common sense recommended by others. From the perspective of sociality, extroverts are more active, social, adapt faster, and are active when working with others. Their thinking patterns are realistic, execution first, and centered on others. Therefore, it can be said that an extrovert is highly other-centered and environment-dependent, and he or she can correctly recognize and make a judgment on the surrounding situation. During the COVID-19 pandemic, it has been presumed that extroverts can actively learn and absorb new knowledge but may require synchronized actions owing to the emphasized relationships with others. The aforementioned results of university students support this hypothesis: The higher the score of extroversion, the higher the coronavirus knowledge, and the more self-defense measures implemented. However, there was a high tendency to go out with others. Therefore, the character of extroversion has the useful side of knowledge acquisition and self-defense, and the opposite effect of not strictly following the stay-at-home order. A recent report on a psychological and behavioral survey on COVID-19 in Japan revealed that extroverts scored highly for infection prevention behavior and mask-wearing behavior [42], which was consistent with the findings above.

Another aspect to note is that people’s behaviors are influenced by how they pay attention to themselves; that is, self-consciousness [29]. Private self-consciousness is a measure of individual differences considering the extent to which they pay attention to those aspects of themselves that are not directly observed by others, such as inner feelings, emotions, and moods. When self-consciousness is high, people monitor themselves, act in harmony with their own will and values, and provide planning in their lives to achieve their goals. This refers to the so-called spirit of self-denial or severity for oneself. Conversely, public self-consciousness shows individual differences in the degree to which they pay attention to the aspects of themselves that others can observe, such as clothes and hairstyles, or their behavior toward others. They refrain from self-centered behavior that may be criticized by the group or use expectations of others and norms of the field as their behavioral standards. Their appearance varies depending on the situation [43]. The results of this study point to the behavioral tendencies of self-consciousness. Concretely, to cope with infection, a person high in private self-consciousness does not easily neglect the infectious disease and positively acquires knowledge about it. More importantly, they take strict self-protection measures. On the contrary, although people who are high in public self-consciousness are interested in the spread of infectious diseases, it cannot be said that they place importance on the risk of infectious diseases. In addition, similar to highly extroverted people, it can be said that they tend to violate the stay-at-home order when invited by others because they focus on how others see them.

### Education of students per the critical factors of behaviors may provide benefits

This survey was designed and conducted to evaluate the KAP on COVID-19 among university students in Japan and investigate determinant factors. Meanwhile, through this quantitative evidence, suggestions about reasonable control of the spread of infections among university students and the entire society, including university managers, expert teams, and policymakers, are expected.

Via the aforementioned analyses, the following can be concluded from the aspects of imposing stricter self-restraint and/or acting more safely:

1. Living in the capital region is associated with higher KAP.
2. Being female is associated with higher KAP.
3. Japanese students exhibit slightly stronger self-restraint than international students.
4. Basic knowledge is more important, compared to advance knowledge.
5. Frequent information acquisition, correct explanations of the information, and willingness to receive anxious information are essential.
6. Extroversion is positive to safer preventative action but negative to self-restraint.
7. Those with high private self-consciousness act more safely, but high public self-consciousness influences strict self-restraint negatively.
8. The strength of self-restraint decreases over time.

The ongoing COVID-19 pandemic has substantial impacts on peoples’ lives accompanied by economic damage. Financial support was provided by governments to individual households or small companies to make living easier and maintain social sustainability during the self-restraint or lockdowns. Within the social surroundings and university measures to COVID-19, campus life, lifestyle behaviors, and economic status have dramatically changed for university students, accompanying other changes in their mental health and well-being [11]. These points are incredible when considering university students. For example, the Ministry of Education, Culture, Sports, Sciences and Technology in Japan created a series of financial support measures for university students, including Emergency Student Support [44].

Instead of focusing on university students’ mental health and well-being, this study focused on their frequent activities, assuming that there was a possibility that young university students may exhibit low adherence to self-restraint and protective action. Contrary to our initial expectations, university students in Japan generally showed a high KAP level. However, when we search the factors that influence KAP, we obtained the findings described above. Therefore, we suggest that universities, media, and the government should consider these aspects of university students, devising publicity and education measures to achieve a more significant education effect. For example, it is particularly beneficial and vital to educate or inform those who are less careful of the current situation, or those who are not cautious enough.

### Comparisons with the KAP COVID dashboard from Johns Hopkins University and a recent report on the comparison of knowledge, precaution practice, and depression among students in South Korea, China, and Japan

Our data in this report come from the survey completed on July 16, 2020. While we are revising this paper, similarly, a group from Johns Hopkins University conducted a global KAP COVID survey in July 2020 and published a dashboard online [45]. Although the KAP COVID dashboard focused on the whole population, and no psychological factors were analyzed, which is different from the current study, it provides results for each country and subgroups (e.g., college/university or graduate school vs. secondary school education or lower), making it possible to compare the results indirectly. The KAP dashboard implied that a high percentage of individuals with a college education or above showed high self-reported prevention behaviors in Japan (mask-wearing, 96%; physical distancing, 74%; handwashing, 92%). These results are similar to our findings from the Japanese university students’ survey described previously and illustrated in **Fig 1**. Furthermore, the dashboard shows that individuals with a college degree or above have better knowledge than the other groups when it comes to three or more symptoms of COVID-19 and no treatment or vaccine at the time of the survey. These results also agree with our findings that university students possess a high level of knowledge. Our results further showed that basic and advanced knowledge levels varied in gender, major subjects, education levels, nationality, residence, extroversion, and self-consciousness from logistic and multiple linear regressions. Moreover, the dashboard showed that 70% of individuals with college education or above accepted vaccines. According to our results, 68.5% of college/university students were willing to use newly developed drugs (**Fig 1**). Therefore, despite the different population target groups, our results agree with results from the KAP COVID Dashboard from Johns Hopkins University, suggesting a high reliability.

A paper on the comparisons of knowledge, precaution practice, and depression of students from South Korea, China, and Japan has been reported more recently [46]. The main difference between this report and the present study is that our study focused on KAP factors (e.g., psychological aspects), while the other study explored the depression symptoms. If we only focus on KAP, the previous report indicates that in all three countries, students showed good knowledge and high levels of COVID-19 awareness, and the Japanese group performed better than the other two considering hand hygiene. They also clarified that females tended to have a higher level of preventative measures than males [46]. These results are in line with our findings discussed above.

### Strength, limitations, and future work

The strength of this study is in its target group—the Japanese university/college students. When we launched the study, to the best of our knowledge, no other studies had been reported to investigate the KAP toward COVID-19 among this target group. We conducted this survey to provide evidence. Meanwhile, unlike other KAP studies, we analyzed the determinant factors, including psychological factors (i.e., extroversion, private self-consciousness, and private self-consciousness) for not only the practices of self-restraint and preventative actions, but also for the knowledge and the attitudes toward information. The factors of knowledge and attitudes toward information have not been analyzed in other studies. Understanding determining factors can help us improve KAP among university students during the COVID-19 pandemic.

This study has certain limitations. It mainly adopts a convenience sampling method, which is a nonrandom and nonprobability selection. Recruitment bias may have occurred, sampling error could not be calculated, as well as the response rate, and the anonymity of participants to each other may have been violated. The samples were limited in number, and they exhibited imbalances in the subgroups. Thus, the results may not sufficiently represent the whole population of Japanese university students. More importantly, with regard to response bias due to spontaneity, only students with high awareness responses to the questionnaire may provide results with favorable evaluations, and these self-reported responses may not be the same for the whole population. Furthermore, this study is cross sectional, and the results are time-dependent. As the COVID-19 situation is changing rapidly, the KAP among university students is also changing. The results reported here represent the situation during the survey period. Additionally, the responses obtained before the emergency declaration lifting are far fewer than those obtained after the lifting, making it impossible to analyze behaviors due to the emergency declaration lifting. Therefore, this should be considered when discussing the results.

As this study can only show the related determining factors, further studies are required to clarify causal relationships between the aforementioned factors and the behaviors/actions by controlling baseline information. Moreover, because this survey is cross sectional, to examine the time-dependent KAP changes and factors, longitudinal studies are also necessary.

## Conclusion

Japanese university students have been inclined toward safety and good health preservation during the COVID-19 crisis. Gender, major subjects, education levels, nationality, residence, private self-consciousness, and extroversion have all been associated with knowledge and attitudes toward COVID-19. Capital regions, high levels of basic knowledge, high information acquisition, and correct information explanations have all contributed positively to preventative action. Non-capital regions, male gender, non-bio-backgrounds, high public self-consciousness, high levels of advanced knowledge, incorrect information explanations, and high extroversion have all contributed negatively to self-restraint. Moreover, self-restraint has decreased with time. The understanding of these factors and trends may help university managers, experts, and policymakers in planning countermeasures that would control the future spread of COVID-19 among university students and Japanese society.

## Supporting information

S1 Data (XLSX)
